# Conserving avian vocal culture

**DOI:** 10.1098/rstb.2024.0139

**Published:** 2025-05-01

**Authors:** Ross Crates, Daniel Appleby, William Bray, Naomi E. Langmore, Robert Heinsohn

**Affiliations:** ^1^ Fenner School of Environment and Society, Australian National University, Canberra, Australian Capital Territory 2601, Australia; ^2^ Research School of Biology, Australian National University, Canberra, Australian Capital Territory 2601, Australia

**Keywords:** adaptive management, animal reintroductions, behaviour, birdsong, captive breeding, population monitoring, species conservation

## Abstract

Over 40% of bird species learn their vocalizations from conspecifics. Avian vocalizations therefore represent one of the most pervasive and quantifiable examples of culturally acquired behaviour that evolves and is maintained within populations through conformity and selection. We review research exploring the loss of vocal culture in wild birds and synthesize how this loss may occur through three processes, defined as erosion/fragmentation, divergence and convergence. We discuss the potential to conserve avian vocal cultures in the wild and in captivity, using the regent honeyeater *Anthochaera phrygia* as a case study. Given the current rates of global biodiversity decline, we predict that more examples of avian vocal culture loss will emerge in the future. There is a need, therefore, for a better understanding of (i) how pervasive loss of vocal culture is in birds; (ii) what factors predispose birds to loss of vocal culture; (iii) the fitness costs of loss of vocal culture, including the population size or density range within which fitness costs may be greatest; and (iv) how vocal cultures can best be conserved or restored. This knowledge could then inform management actions such that the diversity of the world’s birds and their vocalizations can be maintained for generations to come.

This article is part of the theme issue ‘Animal culture: conservation in a changing world’.

## 1. Introduction

Animal cultures are socially learned behaviours that are maintained within populations through information exchange and conformity [1,2]. The array of identified non-human cultures continues to increase, ranging from socially learned migration routes to foraging innovations and antipredator behaviours [3–5]. Avian vocalizations remain one of the most widely recognized and quantifiable examples of animal culture. In contrast to calls, which are often produced innately, the songs produced by Oscine birds are learned [6]. Vocal learning is also known to occur in other avian taxa, including the calls of parrots [7], hummingbirds [8], New Zealand wrens [9] and one suboscine species—the three wattled bellbird *Procnias tricarunculatus* [10]. Vocal learning is pervasive in songbirds (Oscines—over 4000 species across 115 families) and parrots (Psittacines—387 species across 4 families), which together represent over 40% of global avifaunal diversity.

Vocal learning in birds is a complex process that, broadly speaking, occurs in two ways [6,11]. Closed-ended song learners require exposure to conspecific tutors during an impressionable period in early life. After a period of imitation and refinement, the songs of closed-ended learners are crystallized by around 1 year of age, after which point their songs remain largely unchanged [6]. Open-ended learners, including parrots, have the capacity to incorporate new sounds into their vocal repertoire as they age [6]. The key point is that, whether they are open- or closed-ended learners, birds must learn many of their vocalizations, and in order to learn vocalizations that are culturally conforming, birds require opportunities to interact with and learn from experienced conspecific tutors [12].

Avian vocalizations are an excellent example of animal culture and an ideal system for improving our understanding of its form and function [5,13]. Birdsong has evolved to play fundamental roles in territory acquisition and maintenance, conspecific recognition, social cohesion and mate attraction, and it thus has clear links to individual fitness [14–16]. Avian vocalizations are readily quantifiable and approaches to the analysis of acoustic data continue to advance (e.g. [14]). Avian vocalizations can also be studied experimentally both in captivity [17] and in the wild [18], offering great opportunity to deepen our understanding of the processes involved in the formation and maintenance of animal cultures. Bird songs additionally have clear parallels with human languages and are intrinsically linked to human culture and well-being [19,20]. The vocalizations of male and female birds often differ in acoustic structure, enabling analysis of sex-specific cultural transmission [21]. Finally, there is increasing evidence that birds’ songs can serve as a ‘canary in the coalmine’ in a conservation context—a warning signal that wild animal populations may be declining in size and/or density [22–24]. The need to conserve animal cultures such as bird song is therefore increasingly being recognized [25,26].

Here, we review the evidence linking reductions in wild bird population size or density to changes in vocal culture and the implications this may have for conservation efforts. We identify processes by which vocal culture can be lost and how this may be impacted by species’ life histories. We then explore the potential to conserve and restore vocal cultures in both wild and captive bird populations. Finally, we identify key research areas, knowledge from which would help improve our future capacity to conserve threatened bird species, their vocal cultures and potentially important socially learned behaviours in other animals.

## 2. Loss of vocal culture in wild birds

### (a) The evidence for loss of avian vocal culture

The first published evidence that population decline and isolation are linked to a reduction in vocal diversity at both the individual and population levels comes from Dupont’s larks *Chersophilus duponti*. Laiolo & Tella [27] showed that song repertoire diversity in males occurring in smaller populations within a fragmented landscape was lower than in males occurring within larger subpopulations. Similarly, Backhouse *et al*. [28] found male Albert’s lyrebirds *Menura alberti* occurring in smaller rainforest fragments tended to mimic fewer species in their repertoires than males occurring in larger fragments. This cultural depletion is likely to be owing to a reduction in the number of lyrebird tutors in smaller fragments, as Albert’s lyrebirds are understood to learn their mimetic repertoires predominantly from neighbouring conspecifics rather than from model species directly [28]. Additional evidence for cultural erosion/depletion comes from populations that have declined in size through conservation translocations. Reintroduced cirl buntings *Emberiza cirlus* sang different songs and with a smaller repertoire size compared with the larger source population [29], while North Island saddlebacks (*Philesturnus rufusater*) translocated to island refugia underwent cultural bottlenecks that led to the fragmentation and simplification of song cultures [30]. In North Island Kōkako *Callaeas wilsoni*, population size was positively correlated with population-level song diversity and repertoire size [31]. Small, non-native populations of invasive monk parakeets *Myiopsitta monachus* produced less complex learned calls than parakeets within the species’ native range [32]. There are also a small but growing number of reports of species mistakenly learning other species’ songs, presumably owing to a lack of conspecific tutors during the vocal learning phase. The best evidence for this comes from regent honeyeaters (Box 1), but there are anecdotal reports of a prairie warbler *Setophaga discolor* [44] and Florida grasshopper sparrow *Ammodramus savannarum floridanus* [45] mistakenly learning the songs of closely related species. Finally, the vocal cultures of three species of Hawaiian honeycreepers (Fringillidae family) are converging owing to habitat loss. This increases niche overlap between the three species, causing them to learn songs from heterospecifics as well as from conspecifics [46].

Box 1 **Loss of vocal culture in regent honeyeaters**

The regent honeyeater is a Critically Endangered songbird endemic to south-eastern Australia. Widespread habitat loss since European colonization has resulted in the regent honeyeater population suffering severe decline, to the extent that there may now be fewer than 250 mature individuals remaining, spread across their vast extent of occurrence [23]. This decline is probably being driven by a demographic Allee effect: inverse density-dependent population growth owing to lower survival and/or breeding success rates of birds occurring in increasingly smaller flocks [33].

Anecdotal reports of regent honeyeaters ‘mimicking’ the songs of other species emerged in the 1990s [34,35], by which time there were probably fewer than 2000 regent honeyeaters left in the wild. By 2010, there was evidence that the regent honeyeater breeding population had been fragmented into three, each with their own distinctive vocal dialect [36].

A zoo-breeding programme commenced in 1995 and between 2001 and 2020, 285 zoo-bred birds were released in northern Victoria [37]. In 2021, the release location shifted to the greater Blue Mountains, where most of the wild population persists [38]. The new focus is to maximize the density of the wild population to facilitate the assimilation of zoo-bred birds into wild flocks and help overcome the potential demographic Allee effect.

A range-wide population monitoring programme commenced in 2015, with the aim of increasing the quality and quantity of data available to help inform management actions to conserve the wild regent honeyeater population [39]. From this time, we have maintained a database of regent honeyeater song recordings, with concurrent data on the breeding status and success of each male located. These data helped us show how song culture is being lost in regent honeyeaters [23]. Around 12% of wild males have learned the songs of other species [23]. Given that these males often occur in areas of particularly low population density, interspecific song learning is probably caused by a lack of exposure to suitable conspecific tutors in early life, causing isolated males to learn the songs of one of a variety of different species with which they co-occur during their song-learning phase. All available evidence from resighted colour-marked wild birds and zoo-based experiments suggests that regent honeyeaters are closed-ended song learners and they fix their adult-type songs by the time they are 1 year old. The study also showed that a proportion of the population are learning a simplified version of the species-typical song, with fewer syllables, fewer unique syllables and a shorter duration (the ‘clipped Blue Mountains’ dialect), suggesting the species’ song culture is eroding as the population continues to decline [23].

Breeding data showed that males whose songs differed from the cultural norm were less likely to be paired to a female, and those that were paired to a female were less likely to initiate a nesting attempt [23]. Subsequent monitoring has revealed that wild regent honeyeater song culture has continued to simplify; the song dialect that we considered to be the cultural norm in the Blue Mountains between 2015 and 2019 (the ‘typical Blue Mountains’ dialect) was no longer detected in the wild population by 2021 [40]. Concurrently, the fitness costs previously linked to singing the simplified clipped Blue Mountains song have also decreased as this song becomes more common [40].

Our research has also shown that zoo-bred birds have developed their own song culture that is distinct from all wild birds [23]. Because the zoo population was initially founded with nestlings, and juvenile males are crèched together when independent at *ca* one month of age, the zoo population has never had the opportunity to acquire wild regent honeyeater song culture. Instead, young zoo-bred males appear to have learned songs from each other, leading to the crystallization of an adult song reminiscent of the ‘babbling’ calls of juveniles [41].

In response to this finding, since 2020 there has been a concerted effort to adaptively manage the zoo population by teaching juvenile regent honeyeaters the ‘typical Blue Mountains’ wild song culture. This has been achieved by attempting to replicate in captivity the song-learning environment that juvenile regent honeyeaters should experience in the wild, involving (i) playback experiments using recordings of wild males between 2015 and 2019 that produce the typical Blue Mountains song; and (ii) live tutoring using two wild adult males that were recruited to the zoo program in 2019 and that produce the typical Blue Mountains song [42]. Given that the typical Blue Mountains song has since disappeared from the wild, the zoo-bred population is now the sole remaining source of this traditional cultural knowledge. It is hoped that reducing the cultural divide between the songs of zoo-bred and wild birds will help to increase the post-release fitness of zoo-bred birds by reducing the potential for assortative mating between the two cohorts [43].

### (b) Mechanisms leading to loss of vocal culture in birds

What broad lessons can we take from the literature summarized above? The first is that loss of avian vocal culture is a process distinct from cultural evolution or revolution. While evolutions (slowly) or revolutions (quickly) can lead to changes in population-level vocal culture, they do not necessarily lead to an overall reduction in cultural diversity and are not necessarily associated with declines in population size or density [19,47]. In contrast, loss of avian vocal culture is associated with reductions in the size, density and/or connectivity of bird populations and is linked to reductions in the individual- and/or population-level diversity or conformity of learned songs and calls.

**Figure 1.**
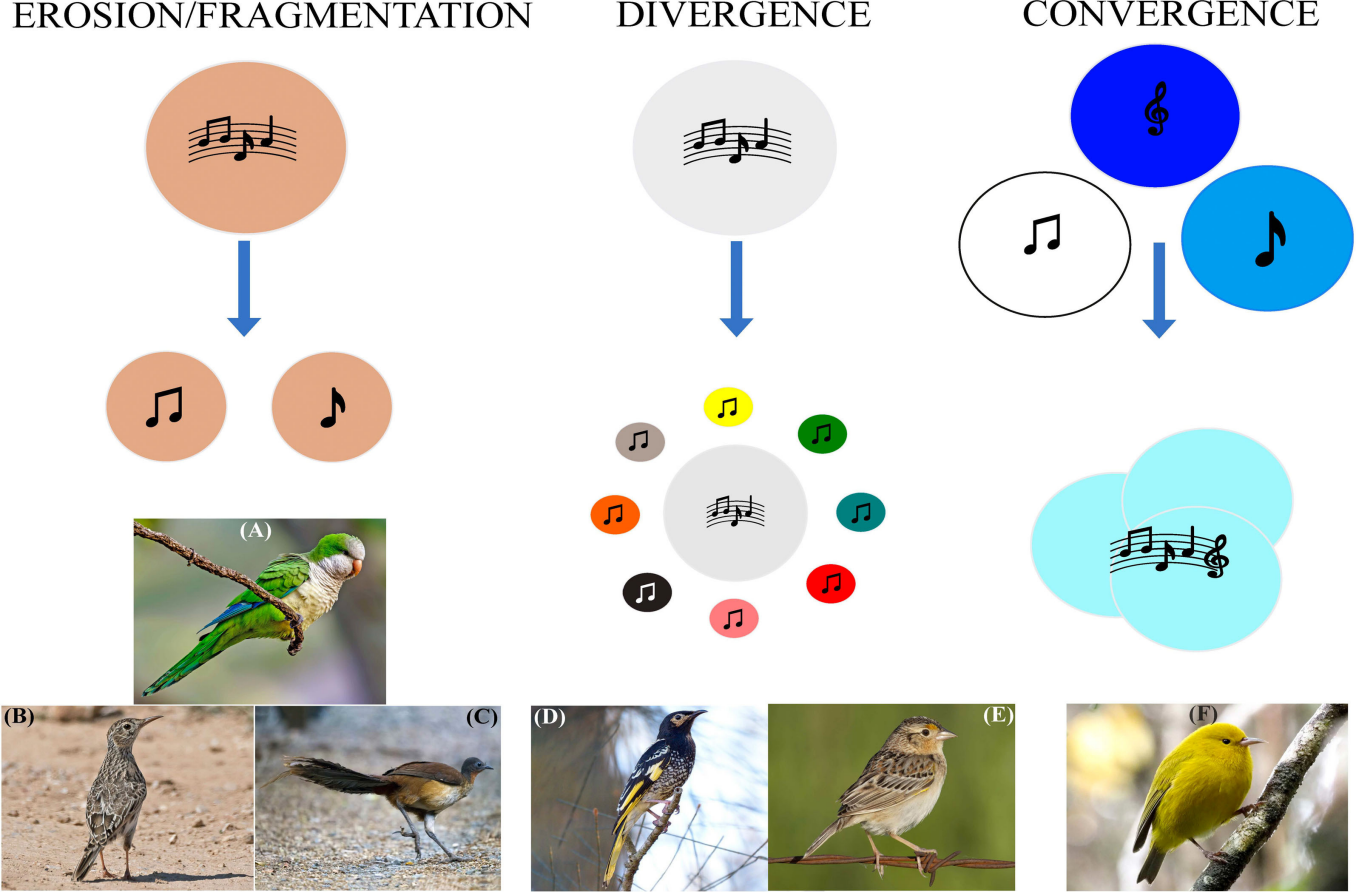
Published examples of loss of vocal culture in birds are grouped into three processes. Circles denote a species’ vocal culture, circle sizes denote repertoire sizes and colours denote species. Erosion/fragmentation of population-level vocal diversity and/or of individual vocal complexity is the most commonly observed process, having been reported in (A) monk parakeets *Myiopsitta monachus* [32], (B) Dupont’s larks *Chersophilus duponti* [27] and (C) Albert’s lyrebirds *Menura alberti* [28], among others. Under a divergence process, some individuals learn one of a range of other species’ songs, leading to diversification of vocal culture at the population level and an overall reduction in the size and/or complexity of the species-typical repertoire. This has been shown in (D) regent honeyeaters *Anthochaera phrygia* [23] and (E) a Florida grasshopper sparrow *Ammodramus savannarum floridanus* [45]. Convergence, whereby multiple species’ songs converge on a common song type, is the least-reported process of avian vocal culture loss, currently only reported in Hawaiian honeycreepers including (F) anianiau *Magumma parva* [46]. Images reproduced under a non-commercial licence agreement with the Cornell Lab of Ornithology. See acknowledgements for image credits.

We suggest that the loss of avian vocal culture can be categorized into three broad processes (figure 1). The most common process we define as ‘erosion/fragmentation’. This process leads to the loss of song diversity at the population and/or individual level in species whose populations are becoming smaller, sparser or more fragmented. Akin to genetic drift [48], erosion/fragmentation may be more pervasive in largely sedentary species, where opportunities for dispersal of individuals with diverse or complex songs between subpopulations—analogous to gene flow at the molecular level—increasingly diminish as those populations become smaller. In this way, vocal culture can be lost through small sampling effects and vicariance.

Process two, defined as ‘divergence,’ occurs in species in which individuals lack opportunities to learn species-specific vocalizations at low population densities. Divergence causes individuals to learn the vocalizations of other bird species they may co-occur with in the landscape owing to a lack of opportunity to interact with and learn from conspecific tutors. This leads to a diversification of vocal culture or a reduction in cultural conformity at the population level. Diversification is distinct from avian vocal mimicry based on the definition of Dalziell *et al*. [49], because (i) individual birds typically learn the vocalization of only one other species as opposed to a range of species; (ii) the sound produced does not change the behaviour of the recipient; and (iii) the sound does not convey a fitness benefit upon the individual producing it [50]. Divergence is best illustrated in regent honeyeaters (Box 1), though anecdotal examples also exist in new world sparrows (figure 1) and warblers. Recent evidence also links the population decline in yellow-naped amazons *Amazona auropalliata* to greater overall acoustic variation through acoustic drift [24]. Because divergence is likely to require very low population density of the focal species and the learning of a broad range of other species’ songs, we predict that divergence is most likely to occur in mobile species including migrants and nomads, or in species that are otherwise unnaturally highly dispersed as a result of severe population decline.

Process three we define as convergence, whereby the songs of multiple, co-occurring species shift towards a single ‘pan-specific’ vocal culture owing to the convergence of closely related species in niche space. Convergence is most likely to be driven by anthropogenic habitat loss, as is the case for Hawaiian honeycreepers [46]. We predict that convergence is most likely to occur in sedentary species occurring in areas of high species richness at the family level. Convergence may therefore be most likely to occur in tropical areas, particularly in species that occupy small territories in densely vegetated environments and therefore rely to a lesser extent on physical interaction to learn culturally conforming songs [51].

### (c) Are there fitness costs associated with the loss of vocal culture?

Given the fundamental links between bird song evolution and individual fitness [15], it seems logical that loss of vocal culture should incur fitness costs. Yet few studies have tested whether or not individuals with depleted or atypical vocal repertoires have lower fitness, or whether loss of vocal culture can contribute to population decline. For example, declines could occur by increasing the challenge of finding mates [52], or by generating reproductive barriers between subpopulations [53]. Male regent honeyeaters whose songs differed from the regional cultural norm were less likely to be paired with a female, and those that were paired were less likely to initiate a nesting attempt [23] (Box 1). Yet this is only correlative evidence, and the relationship may partly be explained by birds with atypical songs tending to occur in areas of particularly low population density, where females are hardest to find. In recent years, we have observed an increase in the proportion of males singing the ‘clipped Blue Mountains’ dialect at the expense of males singing the ‘typical Blue Mountains’ dialect. Concurrently, the fitness costs previously associated with singing the clipped Blue Mountains songs have waned, suggesting that as a new song type becomes more widespread, frequency-dependent shifts in female song preference may ameliorate the fitness costs associated with reduced song complexity [40].

**Figure 2.**
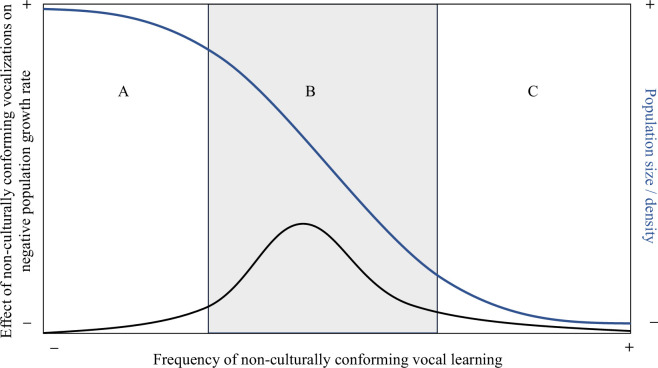
Conceptual relationship between population size/density, frequency of non-culturally conforming vocal learning in birds and its impact on population growth rates. (A) At high population density, non-culturally conforming vocal learning is rare. (B) As population density declines, non-culturally conforming vocal learning increases and females avoid pairing with males with non-conforming/depleted vocalizations. (C) At low population density, females have less mate choice. Non-culturally conforming vocal learning has less impact on population growth rates, but other component Allee effects such as mate-finding contribute to continued population decline.

Could it be that the fitness costs associated with loss of vocal culture only occur within a critical population density range (figure 2)? To select against males with suboptimal songs, females must (i) experience males with both typical and atypical songs; and (ii) have the capacity to choose between them. At natural population densities, atypical song production should not occur because all young birds have opportunities to learn culturally conforming songs from older conspecific tutors. At very low population densities, either all males may sing atypical songs or females may only have a single male to choose from and thus cannot select against them without forsaking breeding altogether. Under this scenario, loss of song culture may only be driving population decline under very specific demographic circumstances. Such circumstances may well be short-lived in declining wild populations, so given the lack of targeted research aiming to uncover such effects in species in which they are most likely to occur, the current lack of empirical evidence of the fitness costs associated with loss of song culture in birds is perhaps less surprising than initially thought.

## 3. Conserving avian vocal cultures

### (a) Conserving and restoring avian vocal cultures in the wild

Evidence from both wild and captive studies shows that vocal cultures can recover naturally in conjunction with population recovery. Population-level song repertoire in translocated cirl buntings recovered to the same level as the source population following an initial decline during population establishment [29]. In zebra finches *Taeniopygia castanotis*, wild-type song culture established *de novo* within four generations of captive birds that were not originally exposed to adult tutors [54]. Where population recovery is challenging to achieve, translocation of individuals with diverse repertoires into culturally depleted subpopulations could help maintain or restore vocal culture. Translocations would, in theory, be most beneficial for largely sedentary species whose populations have been unnaturally fragmented, such as the Albert’s lyrebird [28]. Experiments on wild savannah sparrows *Passerculus sandwichensis* show that song broadcast (playback) could also play a role in the maintenance or restoration of vocal culture [18]. But again, this approach is probably only viable for largely sedentary species and can only practically be implemented on a relatively small scale. Options for conserving song culture in highly mobile species appear to be more limited. One potential approach could be to reintroduce captive-bred individuals as tutors for the wild population [42], but first, we must consider how wild song cultures can be preserved or restored in captive populations.

### (b) Conserving avian vocal cultures in captivity

Evidence from foundational studies of song-learning and our own experiments on regent honeyeaters suggest that avian vocal cultures can potentially be preserved or restored in captive populations through relatively simple changes to husbandry protocols to better reflect natural song-learning environments that species should experience in the wild. Factors that could impact vocal culture in captive populations include the age of founders; it is better to harvest adults that have already acquired culturally conforming vocalizations than eggs or juveniles that are yet to learn them. Consideration should also be given to the spatial distribution of adult tutors and juveniles such that juveniles can at least hear and ideally physically interact with adult tutors during their song-learning phase [55]. Similarly, the ratio of adult tutors to juveniles should be sufficiently high such that juveniles are able to correctly identify adults as tutors rather than other juveniles to prevent the songs of adult tutors from being swamped by juvenile vocalizations.

If playback is to be utilized, consideration should be given to the frequency, amplitude and duration of playback bouts, as well as the number of speakers relative to aviary and cohort size. Species-specific knowledge of the duration of the song-learning phases is also important, as this will affect the age at which juveniles must commence song tutoring, as well as the duration of tutoring required, to ensure that culturally conforming vocalizations are crystallized [56]. Evidence is also increasingly showing the importance of exposing zoo-bred juvenile females to culturally conforming songs—even if they do not produce such songs themselves—given potential impacts on future mate preference [43,57]. For open-ended vocal learners, continued exposure to culturally conforming vocalizations is important to ensure they do not drift [6]. The multitude of factors described here emphasizes the importance of considering how animal cultures can best be maintained in captivity from the *very start* of an *ex situ* conservation program, because these factors will impact fundamental management decisions such as founder identity and the design of breeding facilities. After all, a gram of effort to prevent vocal cultures from being lost in captivity is significantly less costly than the kilo of cure required to restore them [58].

### (c) Conserving avian vocal cultures through reintroductions

Efforts to restore lost vocal culture in zoo-bred regent honeyeaters will soon culminate in the reintroduction of zoo-bred males which produce songs that have been lost from the wild population in recent years through the processes of erosion and divergence. Zoo-bred males are now the only source of cultural knowledge that was present in the wild until 2020 [42]. If the wild population continues to diminish, zoo-bred birds will form an increasingly large proportion of the wild population, raising the exciting possibility that zoo-bred animals could be used to help conserve or restore cultures that have been lost from wild populations. To the best of our knowledge, no reintroduction programs have yet attempted to conserve or restore lost wild cultures using zoo-bred animals as sources of cultural knowledge. Such an approach could prove to be a key way of improving the success of *ex situ* conservation programs in future.

In the context of vocal learning, the ability to use zoo-bred birds to restore song culture to wild populations will be influenced by a number of factors, including the ratio of wild to captive birds. This will, in turn, determine the point at which reintroduction efforts should commence, as well as the capacity to release zoo-bred birds in locations where and at times when wild birds are present, giving zoo-bred animals the best opportunity to assimilate socially into wild flocks. Selection of individuals from the same or similar vocal cohorts/populations has also been suggested as an important consideration to avoid assortative mating during reintroductions and translocations [59]. Alternatively, the reintroduction of juvenile zoo-bred birds could help maintain wild vocal cultures. Releasing juvenile birds into post-breeding flocks could give the young birds the best opportunity to learn culturally acquired behaviours—not just those related to vocalizations but also those related, for example, to movements and antipredator behaviours [3]. Such an approach appears to have made a significant contribution to saving the orange-bellied parrot *Neophema chrysogaster* from extinction [60].

## 4. Conclusions

Subtle and incremental changes to the structure of learned vocalizations can be a natural feature of avian cultural evolution [61] and vocal differences between populations can be important for maintaining social cohesion and local adaptation [5]. However, similar to the loss of many indigenous human languages [62], there is increasing evidence that declines in the size and density of wild bird populations can result in the loss of culturally acquired vocalizations. Given the evolutionary significance of birds’ songs and learned calls [14], there are risks that this loss—if sufficiently pervasive within the population—could magnify the rate of population declines in threatened species [63]. Our current understanding of the potential impact of vocal culture loss on individual fitness and population growth rates is poor.

We suggest that loss of avian vocal culture can occur through three subtly different processes, defined as erosion/fragmentation, divergence and convergence. The susceptibility of different species to loss of vocal culture through these processes will vary, contingent on life-history traits such as mobility, social structure and species-specific intricacies of the song-learning process. Given current rates of avian biodiversity loss [64] and increasing awareness of the potential of vocal cultures to be lost from bird populations, we expect that more examples will come to light in the near future. However, we caution that the species most susceptible to loss of vocal culture are also likely to be among the most difficult species to monitor. This emphasizes the need to increase efforts to improve targeted population monitoring of threatened wild bird species that we predict might be most at risk from loss of vocal culture and to devise effective ways to preserve or restore vocal cultures through conservation actions. In this issue, Whiten & Rutz [65] summarize the growing methodological toolkit that can be used to help achieve this.

There are striking parallels between the ways that cultural and genetic diversity can be lost—and therefore potentially also how they can be conserved. Given that there are very few, if any, studies attempting to conserve avian vocal culture in the wild, borrowing fundamental principles from conservation genetics such as increasing population connectivity and maximizing effective population sizes would be a good place to start [29,66]. Even without empirical evidence from the wild, there is abundant evidence from captive and wild experiments on non-threatened taxa to help guide attempts to conserve vocal culture in species where it is at risk of being lost [18,67].

As has been suggested in recent years, there is a need for greater consideration of animal cultures in conservation efforts [25,26], including those involving zoo-breeding and reintroductions [68]. This can be achieved through greater collaboration between academics and applied conservation practitioners [69], and our work on regent honeyeaters provides a useful example of how this can be achieved within an applied setting [42]. Our experience shows that traditional ‘quantity over quality’ attitudes in zoo-breeding programs can change rapidly given the emergence of robust evidence. Thus, there is great scope for behavioural ecologists, as well as policy makers, to help facilitate this change through greater integration of evolutionary theory and behavioural research into breeding practices, captive experiments and long-term reintroduction strategies [70].

There is still much to learn about the processes involved in the loss of avian vocal culture. How pervasive is such loss in wild bird populations, and what life-history traits most impact species’ capacity to maintain culturally acquired vocalizations? How common are differences in the vocal cultures of wild and captive-bred bird populations, and how could these differences impact the success of animal reintroductions? Can loss of vocal culture indicate that other, more cryptic socially learned behaviours are also being lost from wild populations? What are the fitness consequences of loss of vocal culture, and at what population density range are these costs greatest? Does song erosion/fragmentation constitute a reproductive barrier that prevents gene flow between populations, thereby exacerbating the problems faced by declining populations? Collectively, the answers to these questions will be revealed over the coming decades, helping to inform the implementation of actions to not only minimize global biodiversity loss but also to preserve the emergent properties of biological diversity such as bird song, to which human culture and well-being are so intrinsically linked.

## Data Availability

This article has no additional data.
